# A Legendre–Fenchel Transform for Molecular Stretching Energies

**DOI:** 10.3390/nano10122355

**Published:** 2020-11-27

**Authors:** Eivind Bering, Dick Bedeaux, Signe Kjelstrup, Astrid S. de Wijn, Ivan Latella, J. Miguel Rubi

**Affiliations:** 1 PoreLab, Department of Physics, Norwegian University of Science and Technology, NO–7491 Trondheim, Norway; 2 PoreLab, Department of Chemistry, Norwegian University of Science and Technology, NO–7491 Trondheim, Norway; signe.kjelstrup@ntnu.no; 3 Department of Mechanical and Industrial Engineering, Norwegian University of Science and Technology, NO–7491 Trondheim, Norway; astrid.dewijn@ntnu.no; 4 Department of Condensed Matter Physics, Universitat de Barcelona, Av.Diagonal 647, 08028 Barcelona, Spain; ilatella@ub.edu (I.L.); mrubi@ub.edu (J.M.R.)

**Keywords:** nanothermodynamics, polymers, molecular simulation, single-molecule stretching

## Abstract

Single-molecular polymers can be used to analyze to what extent thermodynamics applies when the size of the system is drastically reduced. We have recently verified using molecular-dynamics simulations that isometric and isotensional stretching of a small polymer result in Helmholtz and Gibbs stretching energies, which are not related to a Legendre transform, as they are for sufficiently long polymers. This disparity has also been observed experimentally. Using molecular dynamics simulations of polyethylene-oxide, we document for the first time that the Helmholtz and Gibbs stretching energies can be related by a Legendre–Fenchel transform. This opens up a possibility to apply this transform to other systems which are small in Hill’s sense.

## 1. Introduction

As we reduce system dimensions from the micro- to the nano-scale, surface properties become increasingly important, and the normal thermodynamic equations (thermodynamic limit properties) cease to apply. Hill [1] proposed a way to restore the structure of ordinary Gibbs’ thermodynamics to deal with small systems. His idea was to introduce an ensemble of small systems, for which ordinary thermodynamics again can be applied. For an in-depth discussion, see also [2]. In Hill’s description, Legendre transforms and Maxwell relations exist, but only at the level of the ensemble of small systems. A single small system, however, does not obey the normal Legendre transforms. A characteristic of small systems is that extensive properties cease to be extensive due to finite size effects, and the thermodynamic potentials depend on the type of environmental control variables, or the ensemble to which they belong. In other words, in general, statistical ensembles are not equivalent for small systems. This striking property is typically observed also in systems with size comparable with the range of the interactions [3,4,5]. Ensemble inequivalence in long-range interacting systems is related to the occurrence of curvature anomalies in thermodynamic potentials, which in this case arise because the interaction energy is not additive. It has been shown that Hill’s approach for small systems can be implemented for long-range interacting systems as well, and that it naturally takes into account the non-additivity induced by the interactions [6,7]. Such a parallelism between small systems and systems with long-range interactions [8,9] indicates that the methods used to describe long-range interacting systems also may find a wider application in the characterization of small systems, and vice versa.

For small systems, the relative size of the fluctuations will be of more significance than for a typical large system. For sufficiently small polymers with a non-linear force-response, one would expect the difference in fluctuations to give rise to size-dependent ensemble deviations. The energy involved in stretching then depends on whether one controls the stretching length or the stretching force. The average force for isometric stretching of a small molecule differs from that of isotensional stretching. In the long polymer limit, they are the same, however, and this has been verified experimentally, computationally, and theoretically. A detailed discussion of this is given by Süzen et al. [10].

We have also studied this problem [11], and verified that the forces were not the same, as predicted from theory. This resulted in a Helmholtz energy for isometric stretching and a Gibbs energy for isotensional stretching for small molecules that were not related by a Legendre transform, which is also known from experiments by Keller et al. [12]. In addition, ensemble inequivalence has been recently highlighted in pulling experiments by Monge et al. [13].

A question therefore arises: is it then at all possible to transform the small system description from one set of variables to another set, like we normally do when we use Legendre transforms? To be more specific: is it possible to transform the Helmholtz energy of a molecule (which describes isometric stretching) into its Gibbs energy (which applies for isotensional stretching)? The aim of this short communication is to show that this is indeed possible.

We shall use our earlier simulation results [11] and verify that the Helmholtz and Gibbs energies for the stretching of a short polymer can be related to each other using the Legendre–Fenchel transform [14], a generalization of the usual Legendre transform, suitable for free energies that exhibit curvature anomalies. This transform has already proven useful in long-range interacting systems displaying ensemble inequivalence [5,15,16], and here it is applied for the first time to a common stretching phenomenon. The Legendre–Fenchel transform reduces to the usual Legendre transform when the Helmholtz energy is differentiable and convex; in the present case, this happens for large polymers. As we precisely show with our numerical simulations, the Helmoltz energy of the considered small polymers in fact present curvature anomalies under certain conditions, making it impossible to use the conventional Legendre transform.

## 2. Method

This section is split into two parts. The first part introduces the model and the computational details, and the second part presents the theoretical method.

### 2.1. Simulation Details

We use the same model as some of us have used previously [11,17] to investigate molecular stretching of poly-ethylene oxide (PEO) on the form CH_3_−[O−CH_2_−CH_2_]_n_−O−CH_3_ in molecular dynamics simulations. It is a united-atom model with each bead representing either a methyl group, a methylene group or an oxygen atom. This model is based on a common model documented in the literature [18,19,20], and has all the standard contributions to the potential energy from bond stretching, bending, and torsion, and includes also the breaking of bonds. It therefore lends itself well to a testing of the stretching energies. In this particular force-field, the standard harmonic bond stretching potential is replaced by a Morse potential

(1) $$U_{\mathrm{bond}}\left(\left\{R_{i},i=1,N\right\}\right)=D_{ij}{\left[1-e^{-\alpha_{ij}\left(r_{ij}-{\bar{r}}_{ij}\right)}\right]}^{2},$$

where the parameters for the dissociation energies $D_{ij}$ are obtained from density functional computations from the literature [21]. The stiffness of the bond is determined by $\alpha_{ij}=\sqrt{K_{ij}^{s}/2D_{ij}}$. Furthermore, the potentials for the bending and torsion of bonds read

(2) $$U_{\mathrm{bend}}\left(\left\{R_{i}\right\}\right)=\frac{1}{2}\sum_{\left\{ijk\right\}}K_{ijk}^{b}{\left[\theta_{ijk}-{\bar{\theta }}_{ijk}\right]}^{2}$$

and

(3) $$U_{\mathrm{tors}}\left(\left\{R_{i}\right\}\right)=\sum_{\left\{ijkl\right\}}\sum_{\left\{c\right\}}K_{ijkl}^{t,c}\cos{}^{c-1}\left(\phi_{ijkl}\right),$$

where *i*, *j*, *k* and *l* are atoms joined by consecutive covalent bonds. $K_{ij}^{s}$, $K_{ijk}^{b}$, $K_{ijkl}^{t}$ are force constants for stretching (s), bending (b) and torsion (t). ${\bar{r}}_{ij}$ and ${\bar{\theta }}_{ijk}$ are equilibrium values for bond stretching and bending, respectively. All force-field parameters were tabulated previously [11]. Non-bonded interactions were not taken into account in the current work, which means that our model polymer is surrounded by an implicit theta solvent. The force field is compatible with the LAMMPS [22] simulation package, that has been used for all of our computations.

The temperature was set to 300 K during sampling, and was controlled by a Langevin thermostat with a relaxation time of 1 ps and a time step of 1 fs. The initial configurations were exposed to a simulated annealing protocol prior to sampling, in an attempt to capture a representative portion of the phase space [17,23]. The presented data are averaged over 5 ns for 200 samples.

### 2.2. Energy Transforms

For the theoretical analysis, consider now an arbitrary polymer with *N* beads. The energy of the polymer is given by

(4) $$H\left(r_{1},\ldots ,r_{N};p_{1},\ldots ,p_{N}\right)=\sum_{j=1}^{N}\frac{p_{j}^{2}}{2m_{j}}+V\left(r_{1},\ldots ,r_{N}\right)\;,$$

where $p_{j}\equiv \left|p_{j}\right|,$$m_{j}$ is the mass of bead *j*, and $V(r_{1},\ldots ,r_{N})$ is the potential interaction. In our previous work [11,17], we gave an explicit expression for the interaction potential with contributions from bond stretching, bending, and torsion. The polymer is controlled either in the isometric ensemble by fixing the end-to-end distance $x\equiv \left|r_{N}-r_{1}\right|$, or in the isotensional ensemble by applying a stretching force $f\equiv \left|f_{N}-f_{1}\right|$. The canonical partition function in the isometric ensemble is

(5) $$Z\left(T,N,x\right)=\frac{1}{\hbar^{3\left(N-1\right)}N!}\int^{\prime }dr_{1}\ldots dr_{N}\int^{\prime }dp_{1}\ldots dp_{N}\exp\left(-\beta H\right)\;,$$

where the end-to-end distance *x* is controlled, by keeping $r_{N}-r_{1}$ constant in the integral over the spacial coordinates. The prime for the spacial integrals indicates this. The prime for the momenta indicates that we keep the center of mass fixed. Furthermore *ℏ* is Planck’s constant and $\beta \equiv 1/\left(k_{B}T\right)$, where $k_{B}$ is Boltzmann’s constant. Because of the symmetry of the system the partition function *Z* depends only on *x* and not on the direction of $r_{N}-r_{1}$. The partition function for the isotensional ensemble is

(6) $$\Delta \left(T,N,f\right)=\beta f\int_{0}^{x_{\max}}dxZ\left(T,x\right)\exp\left(\beta fx\right)\;,$$

where now the stretching force is constant, and $x_{\max}$ denotes the length of the unfolded polymer. The Helmholtz energy is given by

(7) $$F\left(T,N,x\right)=-k_{B}T\ln Z\left(T,N,x\right)\;,$$

and the Gibbs energy by

(8) $$G\left(T,N,f\right)=-k_{B}T\ln\Delta \left(T,N,f\right)\;,$$

in which *x* and *f* are the relevant conjugated variables as usully considered in thermodynamics and statistical mechanics of polymer systems [24]. It follows from Equation (6) that

(9) $$\exp\left[-\beta G\left(T,N,f\right)\right]=\beta f\int_{0}^{x_{\max}}dx\exp\left\{-\beta \left[F(T,N,x)-fx\right]\right\}\;.$$

This makes it possible to calculate the Gibbs energy in the isotensional ensemble from the Helmholtz energy in the isometric ensemble. As the above derivation shows, this transformation is also correct for small polymers.

For sufficiently long polymers, the usual Legendre transform

(10) $$G\left(T,N,f(x)\right)=F\left(T,N,x\right)-f\left(x\right)x$$

is valid. However, we verified in our first paper [11], using molecular dynamics simulations, that for small polymers, the usual Legendre transform is not valid.

Differences in the Helmholtz energy are calculated using

(11) $$F\left(T,N,x_{1}\right)-F\left(T,N,x_{0}\right)=\int_{x_{0}}^{x_{1}}\bar{f}\left(x\right)dx\;.$$

Gibbs energy differences are calculated using

(12) $$G\left(T,N,f_{1}\right)-G\left(T,N,f_{0}\right)=-\int_{f_{0}}^{f_{1}}\bar{x}\left(f\right)df\;.$$

By Equation (9), one may also find the Gibbs energy from $F(T,N,x_{1})$ in Equation (11). With $x_{0}=0$ and $f_{0}=0$, Equation (9) gives

(13) $$\exp\left\{-\beta G\left(T,N,f(x_{1})\right)\right\}=\beta f\left(x_{1}\right)\int_{0}^{x_{\max}}dx\exp\left\{-\beta \left[F\left(T,N,x\right)-f\left(x_{1}\right)x)\right]\right\}\;,$$

where $f(x_{1})$ is obtained by means of interpolation of the isotensional force-elongation curve. From a saddle point approximation to compute the integral in Equation (13), one obtains

(14) $$-G_{\mathrm{LF}}\left(T,N,f(x_{1})\right)=\max_{x}\left[f\left(x_{1}\right)x-F\left(T,N,x\right)\right]\;.$$

The function $F^{*}\left(T,N,f\right)=-G_{\mathrm{LF}}\left(T,N,f\right)$ is known as the Legendre–Fenchel transform [5,15,16] of F(T,N,x) with respect to *x* at constant *T* and *N*.

The Legendre–Fenchel transform is a generalization of the Legendre transform, well known in statistical physics [5,16], and reduces to the latter when the transformed function is differentiable and convex. An important property of the Legendre–Fenchel transform is that it always yields convex functions; thus $-G_{\mathrm{LF}}\left(f\right)$ is convex in *f* at constant *T* and *N*. Furthermore, if $F^{*}\left(f\right)=-G_{\mathrm{LF}}\left(f\right)$ is transformed again, one has

(15) $$F^{**}\left(x\right)=\max_{f}\left[fx+G_{\mathrm{LF}}\left(f\right)\right].$$

Because $-G_{\mathrm{LF}}\left(f\right)$ is a convex function, at points *f* for which $-G_{\mathrm{LF}}\left(f\right)$ is differentiable the above transform (15) reduces to the usual Legendre transform, leading to

(16) $$F^{**}\left(x\right)=f\left(x\right)x+G_{\mathrm{LF}}\left(f\left(x\right)\right),$$

where f(x) is the unique solution to $dG_{\mathrm{LF}}\left(f\right)/df=-x$. Since $F^{**}\left(x\right)$ is simply the Legendre transform of $-G_{\mathrm{LF}}\left(f\right)$, the former is the isotensional Helmholtz free energy. Moreover, due to the properties of the Legendre–Fencel transform, $F^{**}$ is the convex envelope of the isometric free energy *F*, namely, the largest convex function such that $F^{**}\leq F$. Thus, the isometric and isotensional ensembles are not equivalent if *F* does not coincide with its convex envelope $F^{**}$. In mathematical terms, ensemble inequivalence may arise because the Legendre–Fenchel transform is not necessarily self-dual (or involute), that is, $F^{**}\neq F$ when *F* is non-convex. In contrast, the convex envelope $F^{**}$ of *F* has the same Legendre–Fenchel transform as *F*, meaning that ${\left(F^{**}\right)}^{*}=F^{*}$ [16].

We have outlined a method for obtaining the free energy $G_{\mathrm{LF}}$ in the isotensional ensemble from the free energy *F* in the isometric ensemble. This method applies, in particular, when *F* is non-convex. By computing the derivative of $G_{\mathrm{LF}}=G_{\mathrm{LF}}\left(T,N,f(x_{1})\right)$ with respect to *f*, one obtains the force elongation relation

(17) $$\frac{d}{df}G_{\mathrm{LF}}=-x\left(f\right)$$

in the isotensional ensemble. The purpose of this paper is now to test these formulas.

## 3. Simulation Results

**Figure 1:**
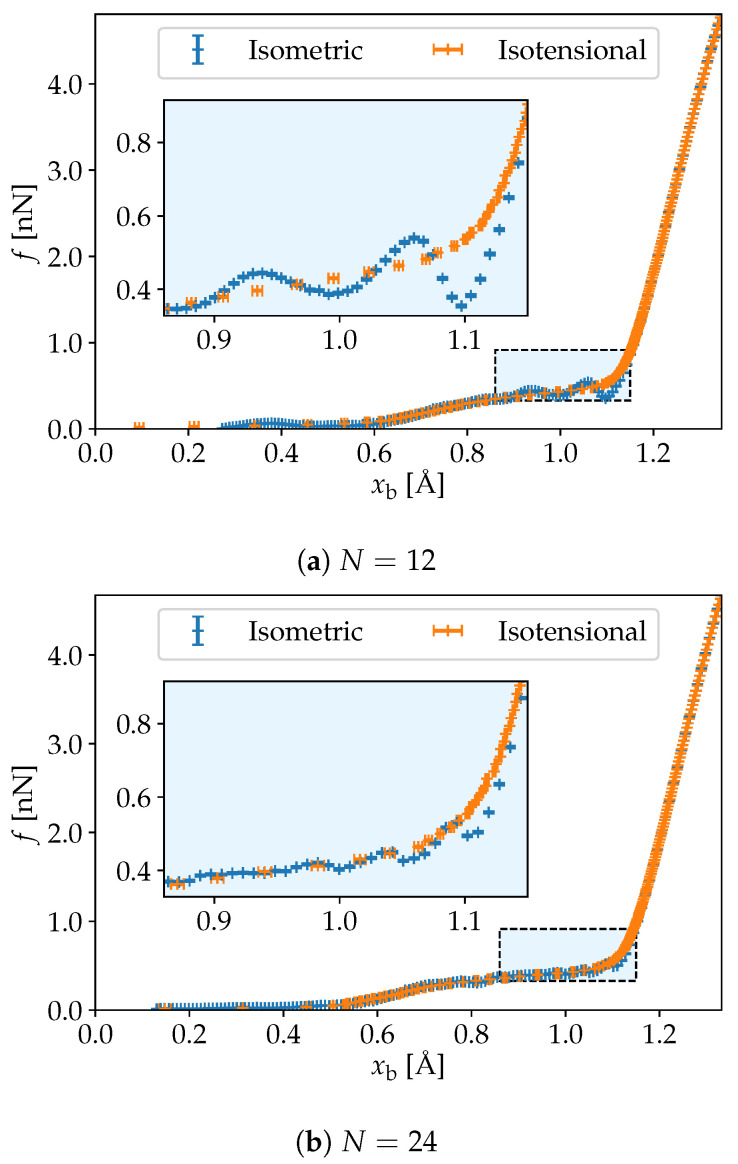

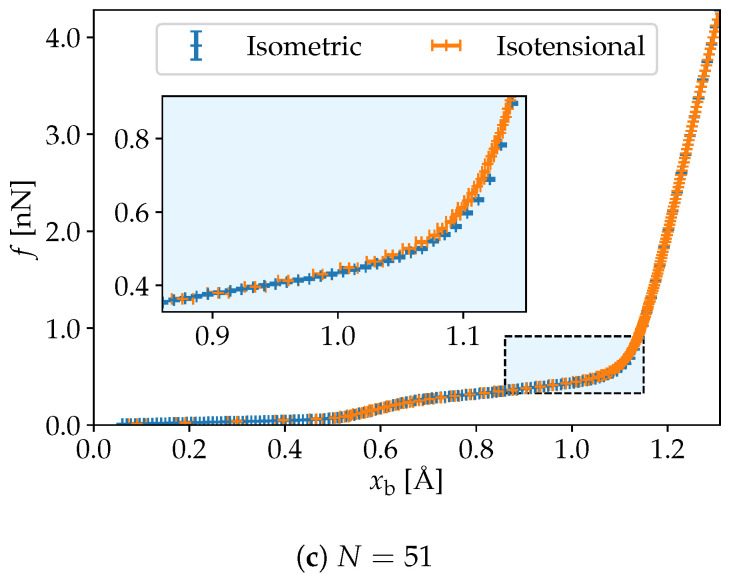
Force as a function of length per bond from isometric and isotensional simulations for chains of poly-ethylene oxide (PEO) composed of N=12, 24 and 51 united atoms. The ensemble inequivalence is most pronounced for the smallest systems.

In the molecular dynamics simulations, one obtains the average force $\bar{f}\left(x\right)={\langle f\left(t\right)\rangle }_{x}$ between the end points in the isometric ensemble. In the isotensional ensemble, one obtains the average distance between the endpoints $\bar{x}\left(f\right)={\langle x\left(t\right)\rangle }_{f}$. The force-elongation curves from the isometric and isotensional ensembles are shown as a function of the length per bond $x_{b}=x/\left(N-1\right)$ for systems of size N=12, 24 and 51 in Figure 1a–c.

It is clear from these figures that the isometric and the isotensional force are different, a fact that is more pronounced for the smaller polymers. In the isometric ensemble, the slope of the curve $\bar{f}\left(x\right)$ is not restricted to be a positive quantity, since the Helmoltz free energy F(T,N,x) is not necessarily a convex function with respect to *x* at fixed *T* and *N*.

In other words, the response function $\kappa^{\left(x\right)}$ defined through

(18) $$\frac{1}{\kappa^{\left(x\right)}}={\left(\frac{\partial \bar{f}}{\partial x}\right)}_{T,N}={\left(\frac{\partial^{2}F}{\partial x^{2}}\right)}_{T,N}$$

can be negative in the isometric ensemble [25], meaning that the associated system configurations minimize the free energy when the average force between the ends of the polymer decreases for increasing elongation. Under these conditions, interactions between monomers tend to separate them from each other, decreasing internal forces required to keep the polymer in equilibrium. We highlight that negative values of $\kappa^{\left(x\right)}$ in this ensemble may be realized because *x* is always kept fixed at a definite value. Furthermore, in the isotensional ensemble, the end-to-end distance fluctuates at constant applied force. In that case, the slope of $\bar{x}$(f) cannot be negative, namely,

(19) $$\kappa^{\left(f\right)}={\left(\frac{\partial \bar{x}}{\partial f}\right)}_{T,N}=-{\left(\frac{\partial^{2}G}{\partial f^{2}}\right)}_{T,N}\geq 0,$$

because internal forces under these conditions do not equilibrate with the external force applied on the polymer.

The points of negative slope in the isometric ensemble can be explained by the torsional unfolding of the molecule. These mechanically unstable modes are not accessible in the isotensional ensemble. As a consequence, we see that the ensemble deviation is most pronounced around $x_{b}=1.1$, which marks the end of the region for torsional unfolding. This was previously discussed in great detail [11]. Prior to this region, around $x_{b}<0.5$, the molecule is twisted helically, and the relation between force and elongation is predominantly linear due to entropic effects. In the last regime, with $x_{b}>1.1$, the molecule is planar, and the force-elongation curve is dominated by the stretching of the individual monomers.

**Figure 2:**
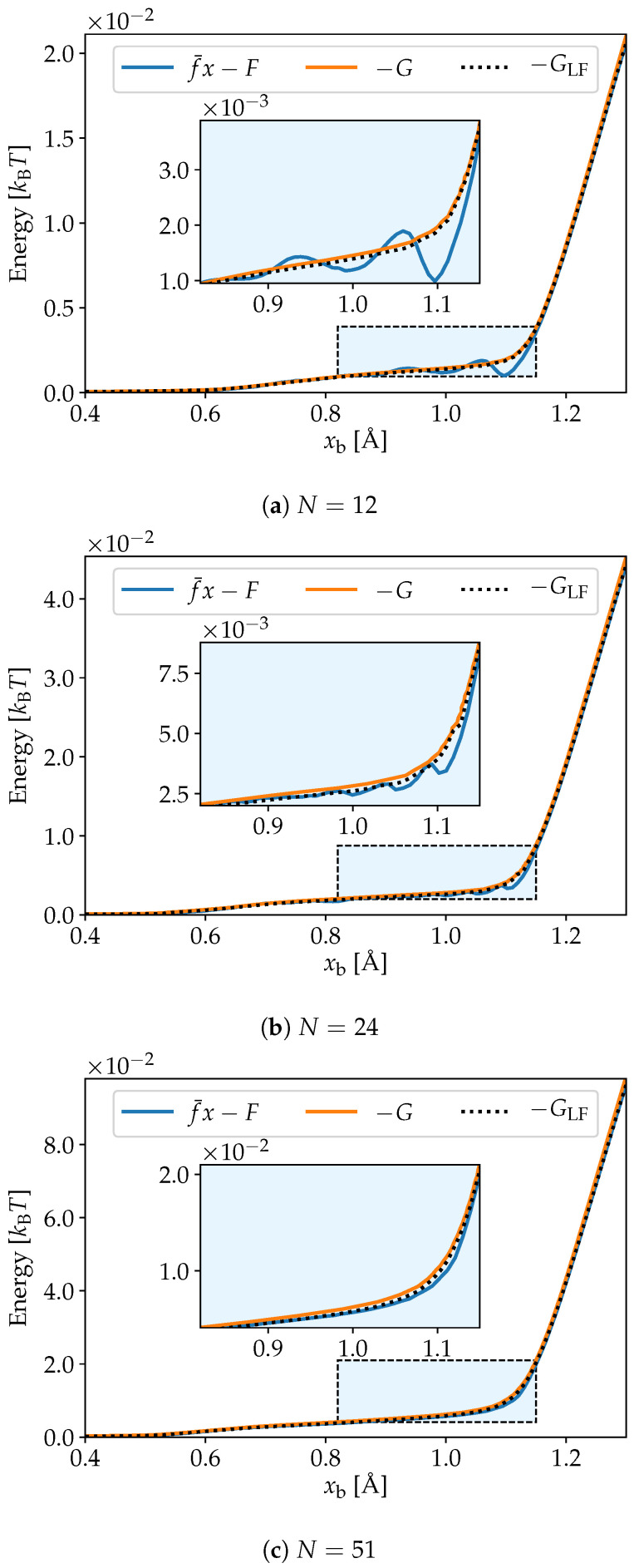
Energy as a function of length per bond for chains of PEO composed of N=12, 24 and 51 united atoms. While the Legendre transform of the Helmholtz energy *F* is different from minus the Gibbs energy *G*, we see that the Legendre–Fenchel transform $G_{\mathrm{LF}}$ is an excellent approximation in all three cases.

Differences in the Helmoltz energy are found from the isometric ensembles by Equation (11), and the Gibbs energy differences are found from the isotensional ensemble by Equation (12). The Legendre–Fenchel transform of the Helmoltz energy is then found by Equation (14). We present these curves for systems of size N=12, 24 and 51 in Figure 2a–c. The Gibbs energy is shown with an orange line, and is compared to the Legendre transform of the Helmholtz energy in blue. It is clear that the Legendre–Fenchel transform of the Helmholtz energy, shown with a black dotted line, gives an approximation to the Gibbs energy that is far superior that of the Legendre transform. We would also like to stress that the Legendre–Fenchel transform is exact in the limit N→∞, since the saddle-point approximation is exact in this limit.

We can see from Figure 2a–c that even for finite *N*, the free energy $G_{\mathrm{LF}}\left(T,N,f(x_{1})\right)$ is in excellent approximation equal to $G\left(T,N,f(x_{1})\right)$. This shows that the exact transformation, which follows from the relation between the partition function, given in Equation (13), as well as the approximate Legendre–Fenchel transform, Equation (14), can be used to obtain the Gibbs energy from the Helmholtz energy for the stretching of small polymers. The curve for $\bar{f}\left(x_{1}\right)x_{1}-F\left(T,N,x_{1}\right)$ is the result of the isometric simulations and differs from the Gibbs energy curves. This shows clearly that the Legendre transform, given in Equation (10), is not valid for small polymers.

**Figure 3:**
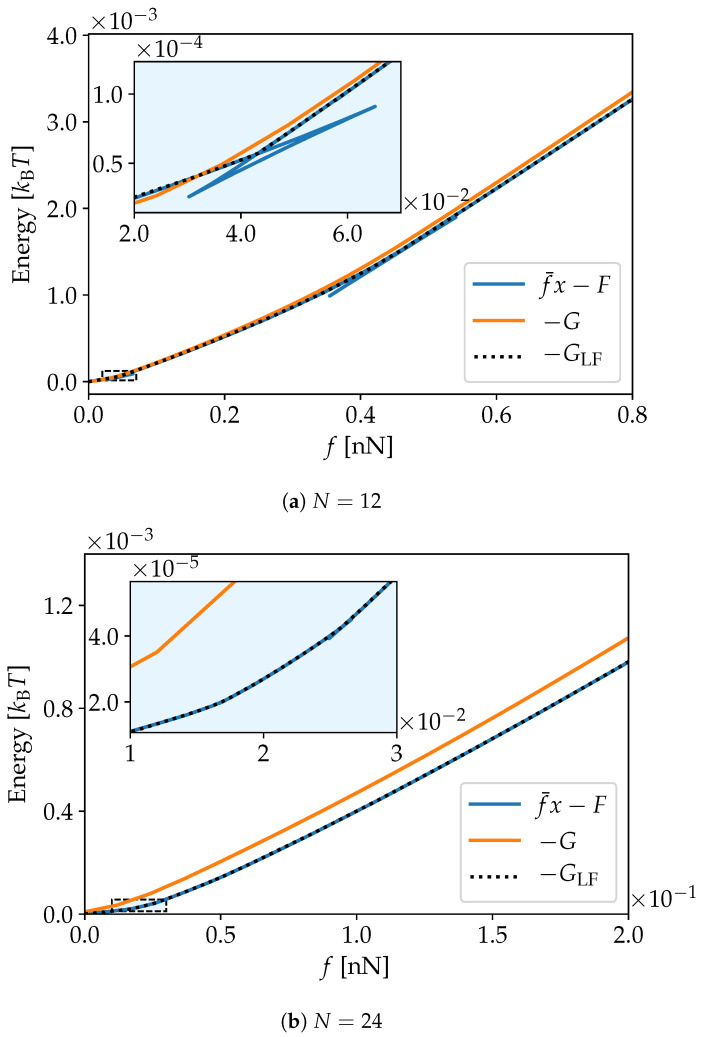
Energy as a function of force for chains of PEO of composed of N=12 and 24 united atoms. The smallest system displays multiple singularities, one of which is emphasized in the insert. Although less pronounced, singularities can be seen also in the system with N=24.

In Figure 3a,b we present the energies from Figure 2a,b as a function of force rather than elongation for systems of size N=12 and 24. As the curves for the Helmholtz energy for these systems are not convex, the corresponding Legendre transformed curves as a function of force is not one-to-one. This is emphasized in the inserts.

**Figure 4:**
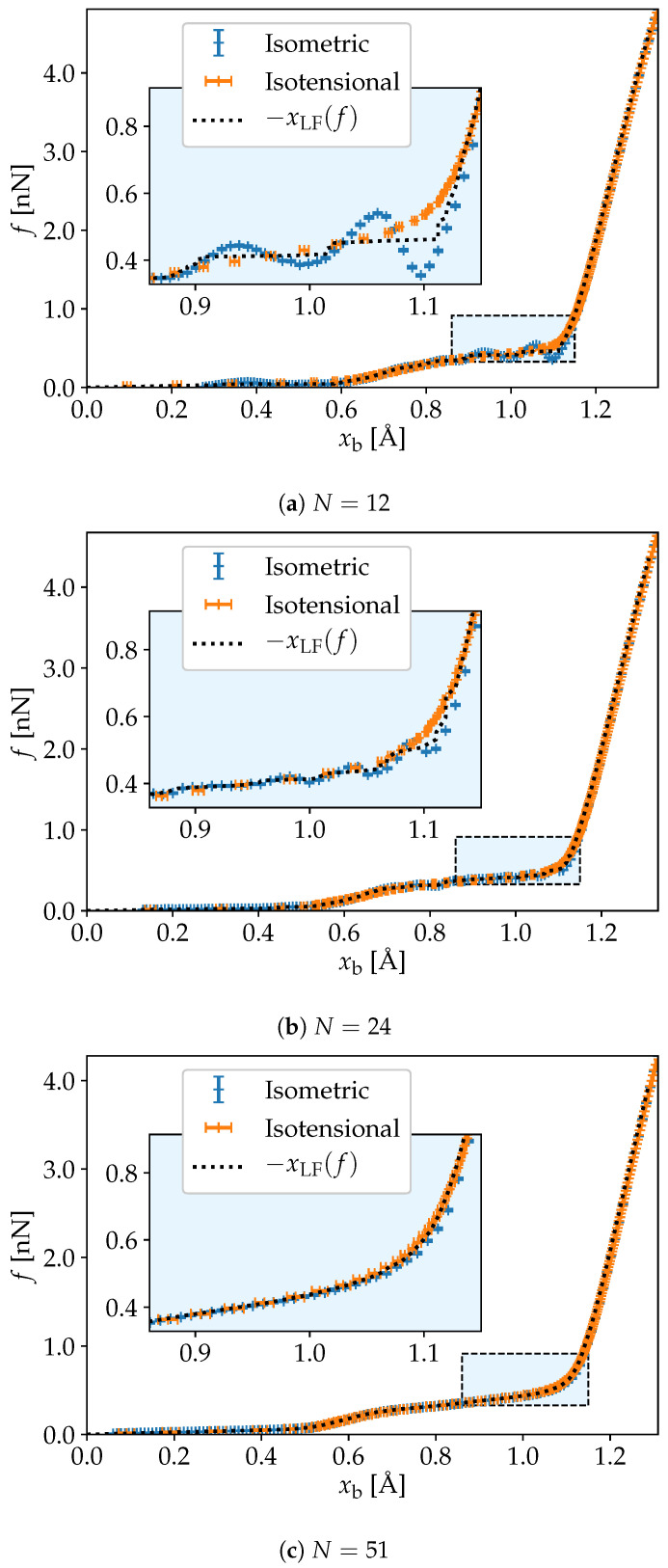
The force-elongation curve $x_{\mathrm{LF}}$ computed from the Legendre–Fenchel transform cf. Equation (17) is compared to the force-elongation curves from Figure 1. We recognize the singular points in $G_{\mathrm{LF}}$ as jumps in $x_{\mathrm{LF}}\left(f\right)$, particularly visible in the smallest system with N=12.

The force elongation relation for the Legendre–Fenchel transformed energy can be obtained by the derivative of $G_{\mathrm{LF}}$ with respect to *f*, cf. Equation (17). This monotonically increasing curve is shown with a black dotted line in Figure 4a–c, with the original force-elongation curves for comparison. It is clear that the Legendre–Fenchel transform is non-involutive for N=12, and that it is involutive for N=51, where it reduces to the Legendre transform [14].

## 4. Discussion and Conclusions

We have analyzed the stretching of small polymers in which the thermodynamic limit cannot be invoked. We have shown that small size contributions to the isometric Helmholtz free energy induce curvature anomalies in this thermodynamic potential, which disappear as the number of beads in the polymer is increased. We described a method employing the Legendre–Fenchel transform to manage these curvature anomalies and obtain the isotensional Gibbs free energy from simulations in the isometric ensemble, in such a way that the states characterized by this free energy are unique.

The Legendre–Fenchel transform in Equation (14) reduces to the usual Legendre transform (Equation (10)) when the free energy F(x) is differentiable and convex in *x* at constant *T* and *N*. Legendre–Fenchel transforms rather than Legendre transforms must be used in particular because F(x) is non-convex [5,16]. As noted previously, the Legendre–Fenchel transform always yields convex functions and therefore, $-G_{\mathrm{LF}}\left(f\right)$ is convex in *f* at constant *T* and *N*. The fact that $-G_{\mathrm{LF}}\left(f\right)$ is convex ensures that the slope of the curve $\bar{x}\left(f\right)$ is non-negative, as required in equilibrium states under fluctuations of the end-to-end distance. Remarkably, this is the case when the free energy F(x) presents a non-convex anomaly in the isometric ensemble. This implies a negative slope in the curve $\bar{f}\left(x\right)$. The Legendre–Fenchel transform maps the states associated with the anomaly into a point *f* at which $-G_{\mathrm{LF}}\left(f\right)$ is non-differentiable. This behavior is exemplified in Figure 3a,b for N=12 and N=24, respectively; in particular in the inserts. Such singularities are not observed for N=51, as F(x) in this case is convex.

We have seen above that the Legendre–Fenchel transform enables us to transform the stretching energy from the isometric to the isotensional ensemble also for small polymers. This removes the limitations set by the Legendre transforms, applicable only in the thermodynamic limit, and opens up a possibility for wider applications. The scheme documented here for molecular stretching energies reduces to the usual Legendre transforms in the thermodynamic limit.
